# Cloud-Based Artificial Intelligence Classification of Common Intracranial Tumors on Magnetic Resonance Imaging

**DOI:** 10.7759/cureus.114682

**Published:** 2026-08-17

**Authors:** Neel Nawathey, Mark A Bachir, Simran Gill, Akshay J Reddy, Ashesh Desai, Ayush Vyas, Rakesh Patel

**Affiliations:** 1 Osteopathic Medicine, Touro University California, Mare Island, USA; 2 Internal Medicine, California Northstate University College of Medicine, Elk Grove, USA; 3 Medicine, University of California, Los Angeles, Los Angeles, USA; 4 Medicine, California University of Science and Medicine, Colton, USA; 5 Medicine, University of California Riverside School of Medicine, Riverside, USA; 6 College of Medicine, Northeast Ohio Medical University, Rootstown, USA; 7 Internal Medicine, East Tennessee State University Quillen College of Medicine, Johnson City, USA

**Keywords:** artificial intelligence, automated machine learning, brain tumor classification, cloud computing, deep learning, glioma, magnetic resonance imaging, medical image classification, meningioma, pituitary tumor

## Abstract

Brain tumors comprise a heterogeneous group of neurological conditions for which timely and accurate classification is important for diagnostic evaluation, treatment planning, and prognosis. Magnetic resonance imaging is the principal imaging modality used to evaluate intracranial tumors; however, image interpretation may be time-consuming and dependent on specialist expertise. This study aimed to develop and internally evaluate a cloud-based artificial intelligence model for classifying glioma, meningioma, pituitary tumor, and no-tumor two-dimensional brain MRI images from a single publicly available dataset using image-level validation. A total of 5,335 images were obtained from a publicly available dataset and divided into training, validation, and testing subsets using an approximately 80-10-10 distribution. The dataset contained 1,400 glioma images, 1,292 meningioma images, 1,362 pituitary tumor images, and 1,281 no-tumor images. Model development was performed using the Google Cloud AutoML image-classification platform in a single-label multiclass configuration. Performance was evaluated using overall and class-specific average precision, precision, recall, precision-recall curves, confidence-threshold analysis, and a multiclass confusion matrix. At a confidence threshold of 0.50, the model achieved an overall average precision of 0.986, precision of 99.2%, and recall of 99.1%. Class-specific average precision was 1.000 for glioma, 1.000 for no-tumor images, 0.986 for pituitary tumors, and 0.973 for meningiomas. Correct classification rates ranged from approximately 99% to 100% across the four categories. These findings demonstrate the feasibility of using an accessible cloud-based AutoML platform for brain MRI image classification. These findings demonstrate strong internal image-level classification performance; however, the inability to confirm patient-level independence, absence of external validation, and use of a single public dataset limit interpretation of the reported metrics. Independent patient-level and multi-institutional validation is required before the model’s diagnostic performance or clinical applicability can be established.

## Introduction

Primary brain and other central nervous system tumors comprise a heterogeneous group of neoplasms with substantial differences in biological behavior, treatment, and prognosis. In the United States, the annual age-adjusted incidence of malignant and nonmalignant primary brain and other central nervous system tumors is approximately 25 cases per 100,000 individuals [1]. Although many primary intracranial tumors are histologically nonmalignant, their location may produce considerable morbidity through compression of surrounding structures, neurological dysfunction, and increased intracranial pressure. Outcomes differ substantially according to tumor type and grade [2], and contemporary classification increasingly incorporates molecular and genetic characteristics in addition to histological findings [3].

Gliomas, meningiomas, and pituitary tumors are among the most clinically important primary intracranial tumor categories. Gliomas include tumors with distinct molecular profiles, growth patterns, and prognoses, and adult diffuse gliomas are classified using markers such as isocitrate dehydrogenase mutation status and 1p/19q codeletion [3,4]. Meningiomas arise from the meninges and are among the most frequently diagnosed primary intracranial tumors, although higher-grade or invasive lesions may require surgery, radiation therapy, or continued surveillance [1,5]. Pituitary tumors arise primarily from adenohypophyseal cells and may produce symptoms through hormonal dysfunction or compression of nearby structures such as the optic chiasm [6]. Accurate differentiation among these tumor categories is important because their management and anticipated clinical courses differ considerably.

Magnetic resonance imaging plays a central role in the detection, characterization, treatment planning, and surveillance of brain tumors. MRI provides excellent soft-tissue contrast and demonstrates tumor location, morphology, enhancement, edema, hemorrhage, mass effect, and involvement of surrounding structures [7,8]. However, interpretation may be challenging because tumors can demonstrate heterogeneous appearances and overlapping imaging characteristics. Differences in scanner type, acquisition protocol, image orientation, and institutional practices may introduce additional variability [8-11].

Cloud-based automated machine-learning platforms may reduce the need for manual neural-network development, specialized programming knowledge, and local high-performance computing resources. In the present study, we developed and internally evaluated a cloud-based artificial intelligence model designed to distinguish among glioma, meningioma, pituitary tumor, and no-tumor brain MRI images [12-21]. The objective was to evaluate whether an accessible AutoML platform could perform this four-category classification task without manual architecture selection or investigator-directed hyperparameter optimization. In the present study, we developed and internally evaluated a cloud-based artificial intelligence image classification model to distinguish among glioma, meningioma, pituitary tumor, and no-tumor two-dimensional brain MRI images obtained from a single publicly available Kaggle dataset. The study evaluated model performance using internal image-level training, validation, and testing partitions without manual neural-network architecture selection or investigator-directed hyperparameter optimization. This investigation was designed as a technical image-classification study and did not evaluate clinical implementation or readiness for clinical use [22,23].

## Materials and methods

The methodology for this study involved the development and internal evaluation of an artificial intelligence model designed to classify brain magnetic resonance images. A total of 5,335 MRI images were obtained from a publicly available dataset on Kaggle [24]. The images were organized according to the diagnostic labels provided with the dataset into four categories: glioma, meningioma, pituitary tumor, and no tumor. The complete dataset included 1,400 glioma images, 1,292 meningioma images, 1,362 pituitary tumor images, and 1,281 no-tumor images. Individual two-dimensional MRI images served as the unit of analysis. Diagnostic labels were obtained directly from the publicly available dataset and were used as provided; the investigators did not independently verify individual labels using histopathology, clinical records, or expert neuroradiology review. Detailed patient-level demographic information and uniform sequence-specific metadata were not available from the source dataset.

The dataset was randomly divided into training, validation, and testing subsets using an approximately 80-10-10 distribution. Of the 5,335 images, 4,269 were allocated to the training set, 533 were assigned to the validation set, and 533 were reserved for testing. The training set was used to develop the model, the validation set was used by the platform to monitor and optimize performance during training, and the testing set was reserved for final internal evaluation after the training and validation processes were completed. Individual two-dimensional MRI images served as the unit of analysis. Detailed patient-level demographic information and uniform sequence-specific metadata were not available in the source dataset. The dataset was partitioned at the image level. Patient identifiers were not available, and patient-level independence among the training, validation, and testing subsets could therefore not be confirmed. Duplicate and near-duplicate images were not independently screened before model development. Consequently, images from the same patient, adjacent slices, or highly similar images may have appeared in more than one subset.

The dataset was divided at the image level. Patient-level identifiers were not available to the investigators, and patient-level independence among the training, validation, and testing subsets could therefore not be confirmed. As a result, images originating from the same patient, adjacent slices from the same examination, or highly similar images may have appeared in more than one subset.

Model training and evaluation were performed using the Google Cloud AutoML image-classification platform. The model was configured as a single-label multiclass classification system, allowing each MRI image to be assigned to one of the four diagnostic categories. The platform automatically performed image feature extraction, model optimization, and hyperparameter selection throughout the training process.

A total training budget of eight node-hours was provided, and model training was completed in approximately one hour and 59 minutes. No investigator-defined neural-network architecture was constructed. The specific network architecture, learning rate, batch size, optimization algorithm, and other internal model-development parameters were not exposed to the investigators by the AutoML platform. The investigators specified the labeled dataset, single-label multiclass classification task, dataset allocation, and training budget, whereas model selection, feature extraction, optimization, and other internal training procedures were controlled by the AutoML platform. The model-search space, augmentation procedures, and complete internal preprocessing pipeline were not exposed to the investigators. No custom neural-network source code was developed for this study.

During testing, the model generated a predicted confidence score for each of the four possible diagnostic categories. The category with the highest score was selected as the predicted classification. A confidence threshold of 0.50 was used as the primary operating point for reporting model performance. Model behavior was also evaluated across a range of confidence thresholds.

Performance metrics

Model performance was assessed using overall average precision, class-specific average precision, precision, recall, precision-recall curves, confidence-threshold analysis, and a multiclass confusion matrix. Average precision was used to summarize performance across confidence thresholds. Precision represented the proportion of positive classifications that were correct, while recall represented the proportion of images within a diagnostic category that were correctly identified.

The precision-recall curve was used to evaluate the relationship between precision and recall across different confidence thresholds. Confidence-threshold analysis was used to determine how changes in the minimum confidence required for classification affected model performance. The multiclass confusion matrix compared the reference category of each MRI image with the category predicted by the model. Correct classifications were displayed along the diagonal, while off-diagonal values represented misclassifications between categories.

Class-specific average precision was evaluated separately for glioma, meningioma, pituitary tumor, and no-tumor images. Representative images from each category were also evaluated to illustrate the predicted classifications and confidence scores generated by the model. This was a descriptive model-development study, and no formal hypothesis testing or comparative statistical analysis between models was performed.

Ethical considerations

Institutional review board approval was not required because this study used only publicly available, deidentified MRI images and did not involve direct interaction with human participants or access to protected health information.

## Results

The artificial intelligence model was evaluated using a test set of 533 brain MRI images representing glioma, meningioma, pituitary tumor, and no-tumor categories. At a confidence threshold of 0.50, the model achieved an overall average precision of 0.986, precision of 99.2%, and recall of 99.1%. These findings indicate that the model correctly classified the majority of images in the internal test set while producing relatively few false-positive and false-negative classifications.

**Figure 1:**
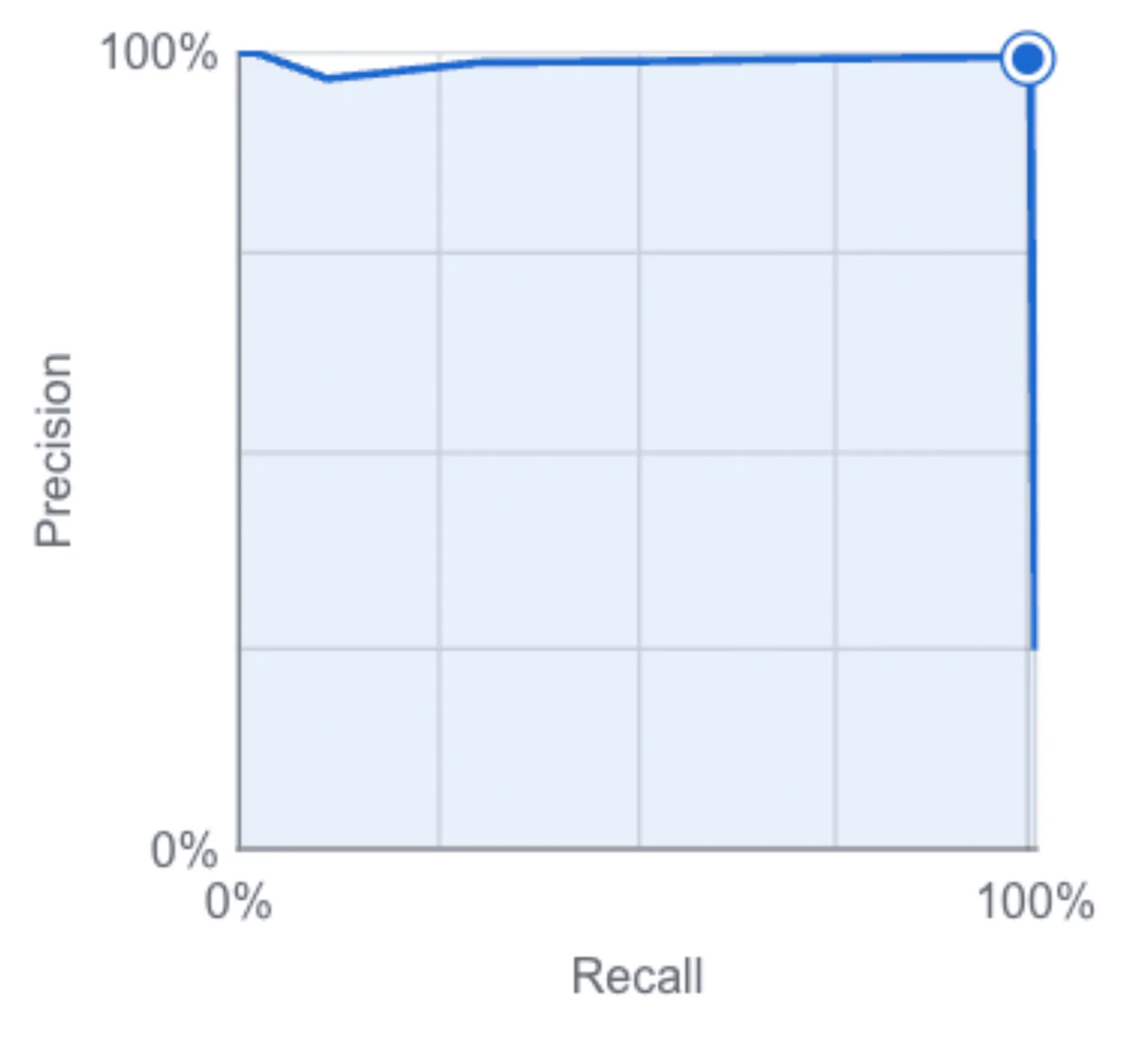
Precision-Recall Curve for the Brain MRI Classification Model The precision-recall curve demonstrates the relationship between precision and recall across different confidence thresholds. The curve remains near the upper-right region of the graph across most thresholds, indicating high precision and recall within the internal test dataset.

The precision-recall curve remained near the upper-right region of the graph across most evaluated thresholds, indicating consistently high precision and recall within the test dataset. Performance declined only at more extreme operating thresholds, as shown in Figure 1.

**Figure 2:**
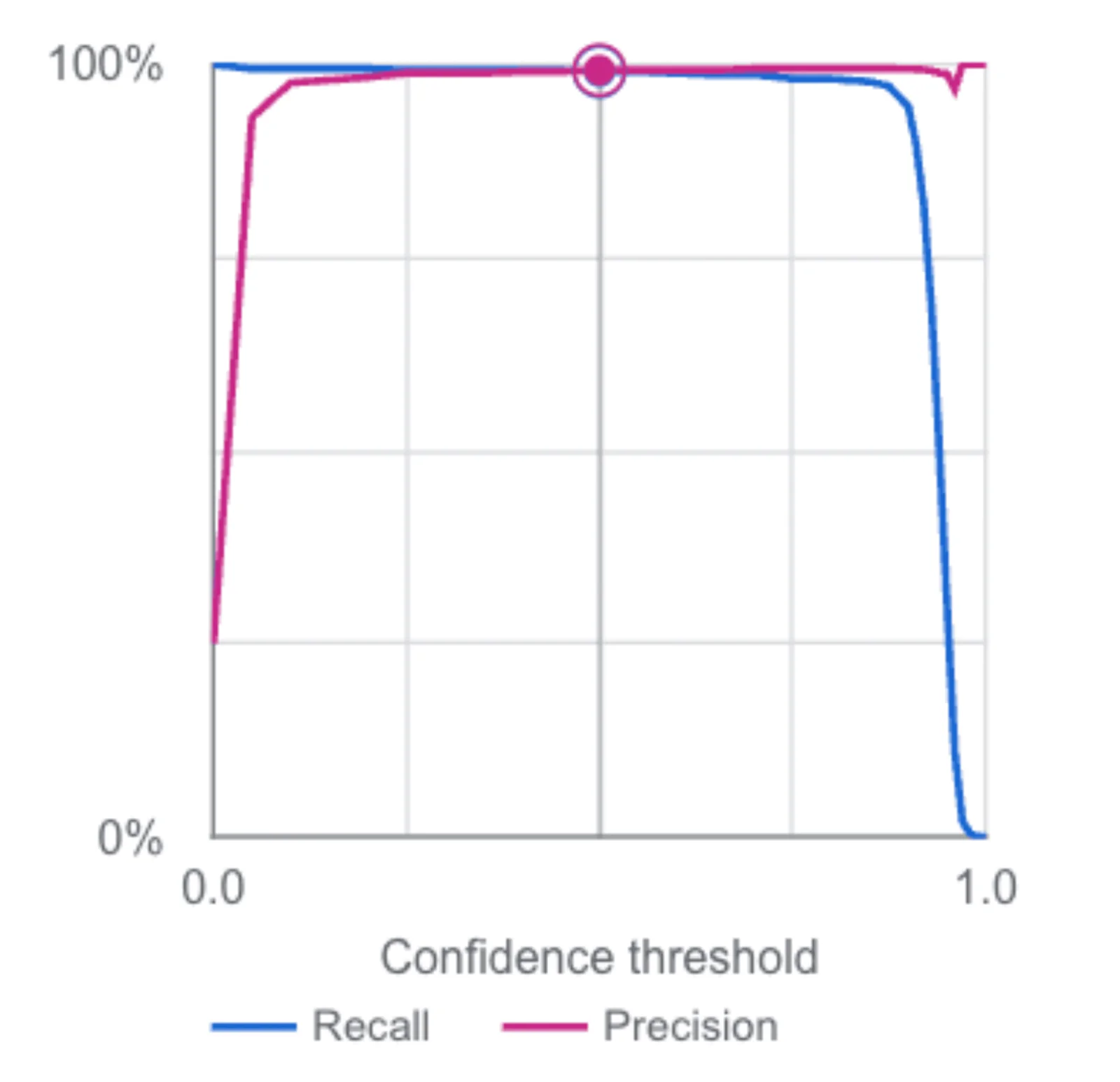
Precision and Recall Across Confidence Thresholds Precision and recall of the brain MRI classification model across varying confidence thresholds. The blue line represents recall, and the magenta line represents precision. At the selected confidence threshold of 0.50, the model achieved 99.2% precision and 99.1% recall.

Confidence-threshold analysis demonstrated the expected relationship between precision and recall. At lower confidence thresholds, recall remained high because the model accepted a greater number of predictions, although precision was slightly reduced. At very high confidence thresholds, precision remained strong while recall declined because fewer images met the required confidence level. At the selected confidence threshold of 0.50, the model achieved a precision of 99.2% and recall of 99.1%, providing a balanced operating point for the present internal evaluation, as shown in Figure 2.

Class-specific evaluation demonstrated high performance across all four diagnostic categories. The model achieved an average precision of 1.000 for glioma images and 1.000 for no-tumor images. Average precision was 0.986 for pituitary tumor images and 0.973 for meningioma images. Although the meningioma category demonstrated the lowest class-specific average precision, performance remained high across all four groups.

**Figure 3:**
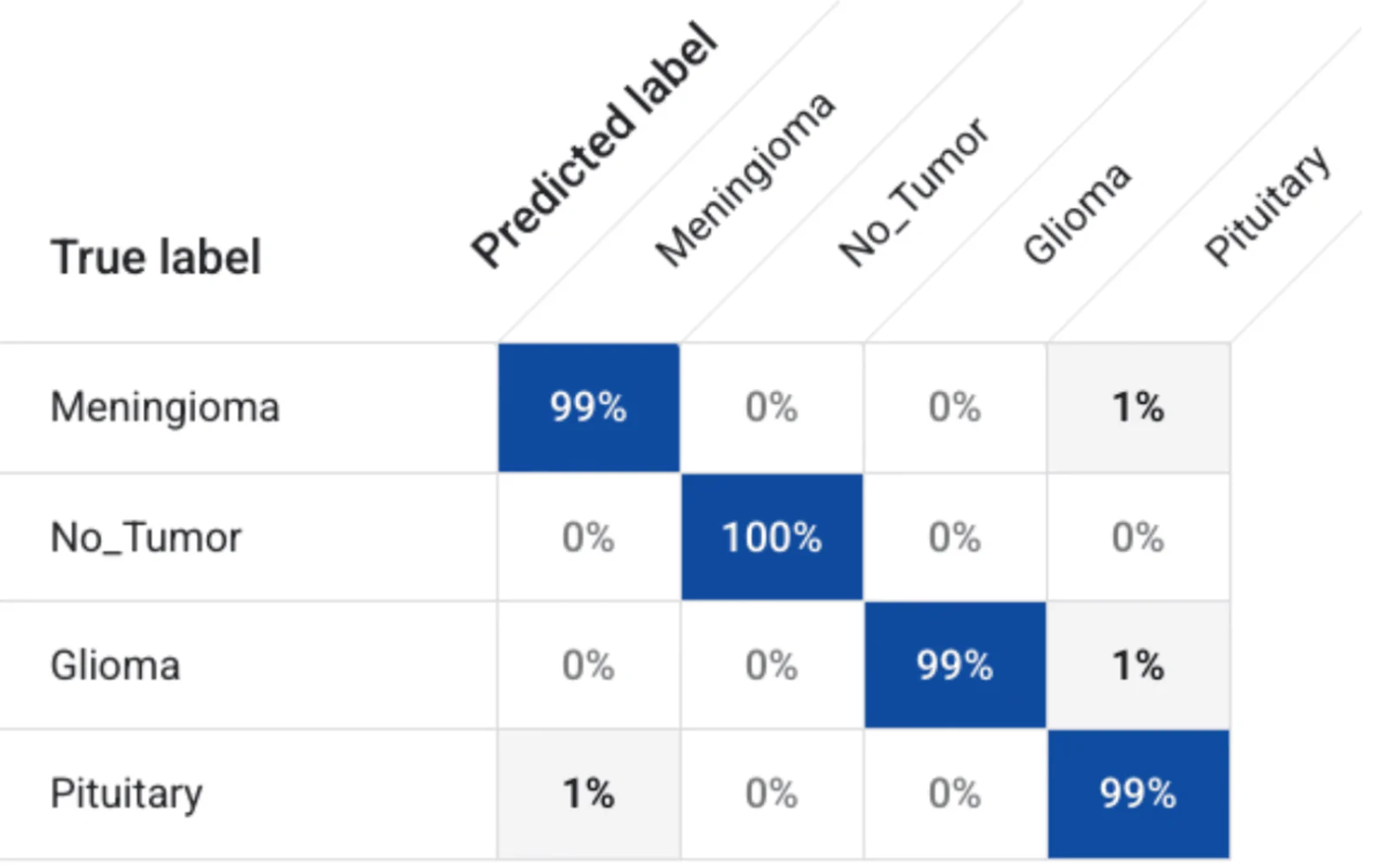
Multiclass Confusion Matrix for Brain MRI Classification Confusion matrix comparing the reference and predicted classifications for meningioma, no-tumor, glioma, and pituitary tumor MRI images. Rows represent the reference labels, and columns represent the model-predicted labels. Correct classifications are shown along the diagonal, while off-diagonal values represent misclassifications between categories. Values are presented as percentages.

The multiclass confusion matrix was used to compare the reference diagnostic category of each image with the category predicted by the model. Approximately 99% of meningioma images were correctly classified, while approximately 1% were classified as pituitary tumors. All no-tumor images were correctly classified, resulting in a 100% correct classification rate for this category. Approximately 99% of glioma images were correctly classified, while approximately 1% were classified as pituitary tumors. Among pituitary tumor images, approximately 99% were correctly identified, while approximately 1% were classified as meningiomas. No meaningful confusion was observed between the no-tumor category and the three tumor-containing categories, as shown in Figure 3.

**Figure 4:**
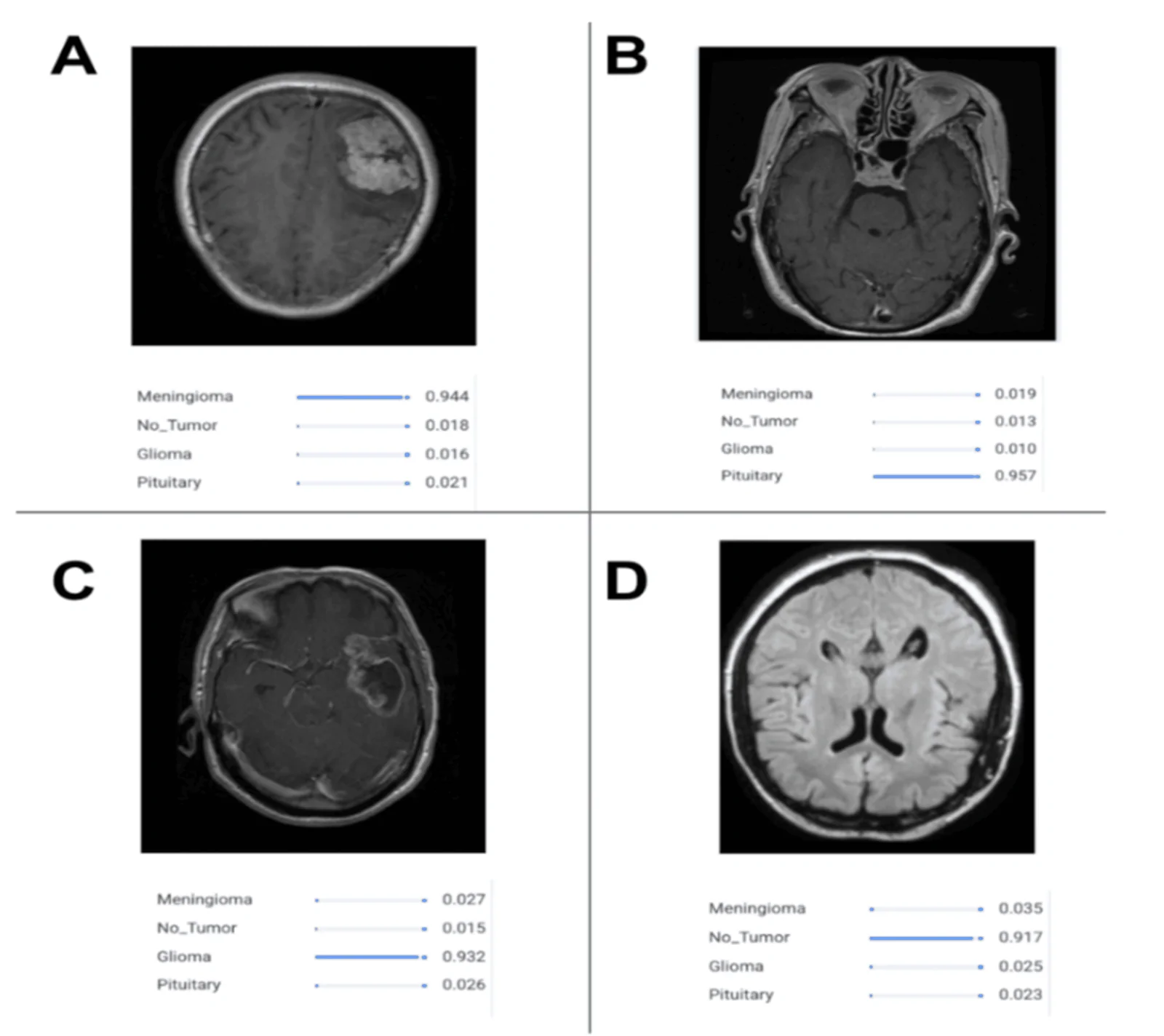
Representative Brain MRI Images Classified by the Artificial Intelligence Model Panel A shows a meningioma image assigned a predicted probability of 0.944 for meningioma. Panel B shows a pituitary tumor image assigned a predicted probability of 0.957 for pituitary tumor. Panel C shows a glioma image assigned a predicted probability of 0.932 for glioma. Panel D shows a no-tumor image assigned a predicted probability of 0.917 for the no-tumor category. The values displayed beneath each image represent the model’s predicted probabilities for the four diagnostic categories. The representative MRI images were obtained from the publicly available Brain Tumor MRI Dataset on Kaggle [24]; classification probabilities were generated by the authors’ model.

Representative MRI images were also evaluated to illustrate the model’s classification behavior. A representative meningioma image received a predicted probability of 0.944 for the meningioma category. A representative pituitary tumor image received a probability of 0.957 for the pituitary tumor category, while a representative glioma image received a probability of 0.932 for the glioma category. A representative image without a tumor received a probability of 0.917 for the no-tumor category. In each example, the correct category received the highest predicted probability, while the remaining categories received substantially lower probabilities, as demonstrated in Figure 4.

## Discussion

The findings of this study demonstrate strong internal image-classification performance using a cloud-based automated machine-learning platform to distinguish among common intracranial tumor categories on magnetic resonance images. The developed model differentiated among glioma, meningioma, pituitary tumor, and no-tumor images with high performance in the internal test set. The precision-recall curve and confidence-threshold analysis further demonstrated that model performance remained high across a broad range of operating thresholds.

Class-specific performance was also high across all four diagnostic categories. The slightly lower average precision observed for meningiomas may reflect heterogeneity in their imaging appearance or overlap with features present in other tumor categories. Meningiomas may vary in size, anatomical location, enhancement pattern, surrounding edema, and relationship to adjacent structures [5,25,26]. However, because image-level error analysis and explainability methods were not performed, the specific factors responsible for individual misclassifications cannot be determined.

The confusion matrix demonstrated that only a small proportion of images were misclassified between tumor categories. These errors may have resulted from similarities in tumor morphology, image orientation, signal characteristics, enhancement patterns, anatomical location, or surrounding structures. Previous research has similarly identified tumor heterogeneity and variability in imaging acquisition as important challenges for automated brain tumor classification [8,10,11,26]. It is also possible that the model relied on dataset-specific characteristics unrelated to tumor biology, including differences in cropping, image borders, contrast, resolution, or acquisition protocols [25,27].

All no-tumor images in the test set were correctly distinguished from tumor-containing images. This finding suggests that the model was effective in separating the included no-tumor images from the three tumor categories within the present dataset. However, the no-tumor category did not include the full range of non-neoplastic abnormalities encountered in clinical practice. The model was not evaluated on infarction, hemorrhage, infection, inflammatory disease, postoperative changes, vascular abnormalities, or other conditions that may resemble neoplastic disease. Therefore, the observed performance should not be interpreted as evidence that the model can distinguish brain tumors from all intracranial abnormalities encountered clinically.

The representative MRI examples further illustrated the model’s classification behavior, with the correct category receiving the highest predicted confidence score for meningioma, pituitary tumor, glioma, and no-tumor images. Although these examples demonstrate how the model generated its predictions, individual correctly classified images do not independently establish robustness or clinical generalizability.

The results are consistent with previous research demonstrating the effectiveness of convolutional neural networks and other deep-learning methods for brain tumor classification [12-21,25,26]. Cheng et al. demonstrated that tumor-region augmentation and image partitioning could improve the classification of gliomas, meningiomas, and pituitary tumors [12]. Deepak and Ameer subsequently showed that features extracted from pretrained convolutional neural networks could be used for brain tumor classification through transfer learning [13]. Other investigators have reported strong classification performance using ensembles of pretrained convolutional neural networks, two-stage feature-level ensemble models, customized convolutional architectures, and vision transformers [14-21].

Akinyelu et al. reviewed machine-learning, convolutional neural-network, capsule-network, and vision-transformer approaches for brain tumor classification and segmentation and found substantial growth in the use of these architectures for MRI analysis [26]. Their review also identified continuing challenges involving dataset quality, image variability, model interpretability, and generalizability. These challenges remain relevant to the interpretation of the present findings.

Ghauri et al. recently developed a cloud-based deep-learning platform for brain tumor classification that used automated optimization, transfer learning, and image augmentation [25]. Their system classified five categories and included testing on additional datasets. The present study differs by evaluating a commercially available no-code AutoML platform for classification of glioma, meningioma, pituitary tumor, and no-tumor images without investigator-directed architecture selection or hyperparameter optimization. Together, these studies support the feasibility of cloud-based computational platforms for brain tumor MRI classification while also demonstrating the importance of evaluating performance beyond an internal data split [25].

Because multiple previous studies have investigated similar multiclass classification tasks, the principal contribution of the present study is not the introduction of glioma, meningioma, pituitary tumor, and no-tumor classification. Rather, this study demonstrates the feasibility of completing this task through an accessible cloud-based AutoML platform without manual neural-network architecture development or investigator-directed hyperparameter optimization. Accordingly, exact model-level reproducibility is limited by the proprietary AutoML environment. Although the dataset source, class definitions, dataset allocation, training budget, classification configuration, and evaluation framework are reported, several internal model-development parameters were controlled by the platform and were unavailable to the investigators. Therefore, the experimental workflow can be repeated using the same publicly available dataset and AutoML framework, but exact reconstruction of the trained model cannot be guaranteed.

The use of a cloud-based automated platform provided several practical advantages. Model development was completed without the need for local high-performance computing equipment or manual construction of a neural-network architecture. The platform automatically performed feature extraction, model optimization, and parameter selection. Similar cloud-based approaches may reduce technical and computational barriers for researchers and institutions with limited programming experience or access to specialized computing infrastructure [25].

However, the automation that improves accessibility also introduces important scientific limitations. The AutoML platform did not disclose the selected neural-network architecture, learning rate, batch size, optimization algorithm, or complete preprocessing pipeline. Consequently, the model may be difficult to reproduce independently, and the contribution of individual development choices cannot be assessed. Transparent reporting of dataset characteristics, preprocessing, model development, and evaluation procedures is essential for determining the validity and reproducibility of artificial intelligence research [22,27].

The model was trained, validated, and tested using images obtained from the same overall public dataset. Although the testing subset was withheld from model training, internal random division does not provide the same evidence of generalizability as evaluation using a fully independent external dataset. Models that perform well on internal data may demonstrate substantial performance degradation when applied to data from different institutions, scanners, acquisition protocols, or patient populations [28-31]. External validation is therefore needed before the performance of the present model can be generalized beyond the source dataset.

The dataset was divided at the image level rather than at a confirmed patient level. Patient identifiers were unavailable, and multiple images from the same patient, adjacent slices from the same examination, or highly similar images may therefore have appeared in both the development and testing subsets. Such overlap could allow a model to recognize patient-specific or examination-specific features rather than generalizable tumor characteristics. Data leakage of this type can produce overly optimistic performance estimates and has been identified as an important threat to reproducibility in machine-learning research [27,28]. A major limitation is that the dataset was divided at the image level rather than at a confirmed patient level. Patient identifiers were unavailable, and duplicate or near-duplicate images were not independently screened. Because patient-level provenance was unavailable, the presence and magnitude of cross-set overlap cannot be determined retrospectively.

The study also evaluated individual two-dimensional images rather than complete MRI examinations. In clinical practice, radiologists evaluate multiple slices, imaging planes, and pulse sequences while incorporating enhancement patterns, diffusion characteristics, prior imaging, patient age, symptoms, medical history, and other clinical information [7-9]. Performance on isolated images therefore does not directly represent performance during complete clinical MRI interpretation.

The model was limited to four diagnostic categories and was not evaluated on other primary brain tumors, metastatic lesions, lymphoma, schwannoma, postoperative changes, treatment-related changes, or non-neoplastic intracranial abnormalities. In addition, the diagnostic labels were obtained directly from the public dataset and were not independently verified using pathology reports, operative findings, longitudinal follow-up, or expert neuroradiology review.

The model was not compared directly with conventional convolutional neural networks, established transfer-learning architectures, radiologists, or other clinicians. Consequently, it is not possible to determine whether the AutoML approach provided superior accuracy, efficiency, or accessibility compared with alternative approaches. Systematic reviews comparing deep-learning systems with healthcare professionals have also found that many published studies lack external validation, prospective evaluation, or direct comparisons performed under equivalent clinical conditions [29,30]. Claims of clinical benefit should therefore remain cautious until the model is assessed against clinicians and alternative computational methods.

No explainability or tumor-localization analysis was performed. Activation maps or related approaches may help determine whether model classifications are based on tumor-containing regions rather than image borders, annotations, scanner characteristics, or other irrelevant features. However, explainability visualizations do not independently establish that a model’s reasoning is clinically valid and should be interpreted together with systematic error analysis and external testing [26,27].

Future studies should use patient-level dataset partitioning and systematic duplicate-image detection before model development. External validation should be conducted using independent datasets representing different institutions, scanner manufacturers, magnetic-field strengths, acquisition protocols, and patient populations [27,31]. Evaluation using complete MRI examinations rather than isolated images would also more closely reflect clinical practice.

Future research should assess model robustness to changes in image orientation, resolution, cropping, brightness, contrast, and image quality. Direct comparison with established architectures such as ResNet, DenseNet, or EfficientNet would help determine whether AutoML provides comparable performance while requiring less technical development. Reporting development time, financial cost, computational requirements, and required programming expertise could further clarify the practical advantages and disadvantages of the platform [25].

Comparison with radiologists or trainees could determine whether artificial intelligence assistance improves diagnostic accuracy, confidence, or interpretation time. Prospective evaluation would also be necessary to determine how model predictions influence clinical decision-making and patient outcomes. Prior reviews of medical-imaging artificial intelligence have emphasized that high retrospective classification performance alone does not establish clinical effectiveness [29,30].

Overall, the developed model demonstrated strong internal performance in distinguishing among glioma, meningioma, pituitary tumor, and no-tumor MRI images. These findings demonstrate that a cloud-based AutoML platform can be used to develop and internally evaluate a multiclass brain MRI image-classification model. However, the reported performance should be interpreted strictly within the context of a controlled public dataset and internal image-level validation rather than as evidence of patient-level diagnostic accuracy, clinical effectiveness, or readiness for implementation.

## Conclusions

This study demonstrates that a cloud-based AutoML platform can be used to develop and internally evaluate a multiclass brain MRI image-classification model. The findings suggest that accessible automated platforms may reduce some of the technical and computational barriers associated with artificial intelligence model development. However, the reported performance should be interpreted strictly within the context of a single publicly available dataset and internal image-level validation. Patient-level partitioning, duplicate-image assessment, external validation, and evaluation using clinically relevant comparator conditions are necessary before conclusions regarding diagnostic accuracy, generalizability, or clinical utility can be made. Although cloud-based artificial intelligence may eventually support brain MRI analysis, it should not replace radiological interpretation, complete clinical evaluation, or pathological confirmation.
